# Acknowledgement

**Published:** 2024-01

The biennial conference, ICON of Indus Hospital & Health Network (IHHN) is a time of spreading knowledge and celebrating our accomplishments through multiple activities including publications. For the 7th Biennial ICON, January 2024, various submissions for publication of our scholarly work were received as original articles, reviews, case reports, and editorials. The work captured in this edition underwent a rigorous peer review process by 53 reviewers - a completely volunteer effort of our esteemed reviewers, for which IHHN and the journal are indebted. Their keen insight and expert opinions made it possible for us to successfully launch this special edition. A sincere thank you again to all our reviewers and administrative support for their generous contributions. Names in italics are of those who did multiple reviews.

***Recognizing the Contribution of Our Reviewers***

**Table:** 

Reviewers from the North and South Campuses of IHHN	Reviewers from various National and International Institutes
Abdul Qaseem Khan	*Adil Akhtar*
*Ahmed Shoaib*	*Afreen Sadia*
Ahmer Hamid	Afsheen Raza
Aisha Wali	Ahsan Maqbool Ahmed
Ammad Fahim	Amna Subhan
Ayesha Shoaib	*Ayesha Aziz*
Faisal Usman	Bushra Ahmed
Faridah Amin Tejani	Farheen Imran
Haroon Ayub	*Hassan Mumtaz*
Javeria Aijaz	Javaid Khan
*Mariam Aftab Chaudhry*	Kauser Hanif
*Mohammad Imran Shekhani*	*Maham Zahid*
Muhamad Zeeshan	Maida Umar
Muhammad Fareeduddin	Mehroze Zamir
Muhammad Nizam Uddin	Muhammad Saleem
Naveed Ahmed	*Naeem Jabbar*
Neelum Mansoor	Nighat Jamal
*Nida Ghouri*	Noorulain Fareed
Saima Ali	Rabia Mushtaq
Saima Saeed	*Saba Khan*
Salima Mehdi	Sahar Mazhar
Sayyeda Reza	Sher Wali
Shazia Jamil	*Shiyam Sunder Tikmani*
Sohail Akhtar	Sumaira Naz
Surhan Badar	*Tahira Ezra Reza*
*Syed Ghazanfar Saleem*	Uzair Naru
Taarif Hussain Warraich	
Urooj Quareshi	
Wasfa Farooq	
Wasim Akhtar	
*Zaira Rehman*	

